# Structured Modeling and Analysis of Stochastic Epidemics with Immigration and Demographic Effects

**DOI:** 10.1371/journal.pone.0152144

**Published:** 2016-03-24

**Authors:** Hendrik Baumann, Werner Sandmann

**Affiliations:** 1 Department of Applied Stochastics and Operations Research, Clausthal University of Technology, Clausthal-Zellerfeld, Germany; 2 Department of Computer Science, Saarland University, Saarbrücken, Germany; Shanxi University, CHINA

## Abstract

Stochastic epidemics with open populations of variable population sizes are considered where due to immigration and demographic effects the epidemic does not eventually die out forever. The underlying stochastic processes are ergodic multi-dimensional continuous-time Markov chains that possess unique equilibrium probability distributions. Modeling these epidemics as level-dependent quasi-birth-and-death processes enables efficient computations of the equilibrium distributions by matrix-analytic methods. Numerical examples for specific parameter sets are provided, which demonstrates that this approach is particularly well-suited for studying the impact of varying rates for immigration, births, deaths, infection, recovery from infection, and loss of immunity.

## Introduction

Epidemic processes are particularly important population dynamics describing the outbreak and spread of infectious diseases. Mathematical models are in widespread use for analyzing and predicting the time evolution of populations. While in the most classical models the total population is closed and of constant size, generalized and extended versions incorporate demographic effects, open populations, and variable population sizes. Deterministic models described by ordinary differential equations (ODEs) have the longest tradition and under certain circumstances they provide suitable approximations. But epidemic processes are often substantially governed by random effects. Stochastic models, especially Markov chains, are then more appropriate. We refer to [1–3] for the general background on Markov chains where [1] focuses on biological including epidemic processes, to [4–6] for introductory texts on epidemic modeling and related stochastic methods, and to [7–9] for extensive surveys of diverse epidemic models. Deterministic and stochastic models are compared in, e.g., [10–12].

Essentially, three regimes of stochastic epidemic modeling are prevalent and well established. With discrete-time Markov chains (DTMCs), populations are represented by nonnegative integers and time is divided into units such as days, weeks, months, or years. In continuous-time Markov chains (CTMCs), the time scale becomes continuous. Stochastic differential equation (SDE) models obey continuous time scales and state spaces, similarly as deterministic ODE models. CTMCs can be approximated by DTMCs and by SDEs. Likewise, also deterministic ODE models can be viewed as approximations of CTMCs. We shall focus on CTMCs. In fact, time evolves continuously and populations appear in discrete quantities.

We consider generalized versions of stochastic SI, SIS, SIR, and SIRS epidemics where the individuals in the population are classified according to the standard terminology in epidemic modeling as susceptible, infective and removed (or recovered). At any time, the state of the corresponding multi-dimensional CTMC is described by the vector consisting of the number of individuals of each epidemiological class. In our model versions we incorporate births, deaths and immigration of individuals of any class where we conceptually distinguish between births and immigration, because immigration rates are typically constant and independent of the population size, whereas births rates depend on the population size and are usually proportional to it. This makes an important difference. With birth rates proportional to the population size (or in some other way requiring the presence of at least one individual) the underlying Markov chains have absorbing states such that quasi-stationary distributions and the time to extinction or the duration of the epidemic are of major interest. With constant immigration rates, the Markov chains are non-absorbing and the disease will not die out forever. Then, if the Markov chain approaches a stochastic equilibrium, equilibrium probability distributions are essential. Of course, a birth and an immigrating individual have the same effect as they increase the population of the corresponding class by one. Therefore, despite their important conceptual difference and the implications on the behavior of the resulting CTMC, they can be modeled by a single state transition type occurring at a combined linear rate with a constant term corresponding to immigration and a term proportional to the population size corresponding to births.

Some features of our models have previously appeared in the literature. For instance, [13, 14] consider SI models with births (rather than immigration) into the susceptible class where the birth rate is proportional to the number of susceptibles present in the population. While in [14] deaths of both susceptibles and infectives occur at rates proportional to the number of individuals of the corresponding epidemiological class, in the version of [13] only infectives can die. Note that in these papers a death of an infective is referred to as a removal, a term that we shall reserve for SIR(S) models with (temporary) immunity. Also [15] deals with an SI model like in [14] but with the birth rate of susceptibles proportional to the total population size. Similarly, [16] studies SI, SIS, SIR, and SIRS models where the SI model is the same as in [15]. In the SIS model additionally the recovery of infectives that then immediately become again susceptibles is included with a recovery rate proportional to the number of infectives. The SIR model accounts for immunity in that recovered infectives do not become again susceptibles but removals from the epidemic. They die at a rate proportional to the number of removals. In the SIRS model the immunity is only temporary and can be lost at a rate proportional to the number of recovered individuals. Of course, in all cases infection of susceptibles is possible. The SIS model of [16] is also considered in [17] but with a different infection rate.

In all these models, due to births rather than immigration there are absorbing states such that the epidemic ultimately dies out. Consequently, the cited works provide approximations of the quasi-stationary distribution and the time to extinction, or the mean duration of the epidemic. Obviously, if one considers the whole world there is no immigration from outside, but in reality epidemics often need to be studied for local moderate-sized sub-communities within a larger community from which there is usually indeed an importation of infections due to the immigration of infected individuals, which is a major underlying rationale behind our models and corroborates their practical relevance.

Models that are in some respects close to ours, though deterministic, are the SIS model and the SIR model in [18]. They comprise a constant flow of new members into the population of which a fraction is infective, and a natural death rate proportional to the population size. Therefore, as in our stochastic models, the infection cannot be eliminated permanently. Furthermore, it is shown in [18] that the deterministic models admit a single asymptotically stable equilibrium. Similarly, considering deterministic models of epidemics for networks with demographics, [19, 20] derive asymptotically stable equilibria. More specifically, for SIS models on networks the basic reproduction number *R*_0_ is obtained and it is shown that for *R*_0_ < 1 there exists a globally asymptotically stable disease-free equilibrium, while for *R*_0_ > 1 there is a globally asymptotically stable endemic equilibrium. In particular, it is shown that demographics indeed has a strong impact on the basic reproduction number and is important with regard to epidemic propagation between communities, see [19, 20] for the details. However, we neither consider epidemics for networks nor deterministic models but stochastic models with immigration and demographic effects. In stochastic models, there is no single equilibrium state but an equilibrium probability distribution of the underlying CTMC. Consequently, we aim at obtaining the equilibrium distributions of our models.

If explicit analytical solutions are not available, computational approaches are required and often stochastic simulation is applied, but even when using advanced methods [21, 22] statistical analysis of steady-state measures by stochastic simulation is inherently costly, in particular if the interest is not only in expectations of population sizes but in probability distributions. Accurately obtaining whole probability distributions by stochastic simulation requires enormous computational efforts since stochastic simulation actually is a computer-based statistical estimation procedure. It consists of generating many independent sample paths for building sufficiently small confidence intervals in order to get statistically reliable results. Clever numerical solution of Markov chains is an alternative computational approach that becomes particularly useful and efficient in case of suitably structured models [23–33].

We shall structure our stochastic epidemic models such that they correspond to level-dependent quasi-birth-and-death (LDQBD) processes, that is, CTMCs with multi-dimensional state space and block tridiagonal generator matrix. For this purpose, first of all a suitable ordering of states is required. For computing the equilibrium distributions we then invoke the matrix-analytic algorithm invented in [24].

Hence, the contribution of the present paper is threefold. Firstly, we introduce stochastic epidemics with births and deaths as well as immigration of individuals of all epidemiological classes involved such that the underlying CTMCs become non-absorbing. Secondly, we show how to model them as LDQBD processes. Thirdly, based on the generator matrix structure provided by the LDQBD process modeling approach, we apply efficient matrix-analytic methods for their solution, which enables us to compute whole equilibrium probability distributions rather than only expectations.

The necessary background on LDQBD processes and their matrix-analytic solution is given in the following section. Subsequently, we present the considered stochastic epidemics and show how they can be efficiently modeled as LDQBD processes. Then we provide numerical examples for specific parameter settings.

## Methods

### LDQBD processes and matrix-analytic solutions

Before introducing our specific stochastic epidemic models, we present the matrix-analytic framework that we shall apply to structure these epidemic models as LDQBD processes and to solve efficiently for their equilibrium distributions. Our description is focused on continuous-time LDQBDs, but the discrete-time case is similar.

Consider a CTMC with infinite multi-dimensional state space. If the state space can be partitioned into disjoint *levels*, consecutively numbered such that transitions are only possible between states belonging to the same or adjacent levels, then the CTMC is said to be a quasi birth-and-death (QBD) process and its generator matrix is block tridiagonal [31, 32]. The partitioning of the state space can be expressed by the disjoint union $S=S^{\left(0\right)}\cup S^{\left(1\right)}\cup S^{\left(2\right)}\cup \cdots$ with $S^{\left(i\right)}\cap S^{\left(j\right)}=\emptyset$ for *i* ≠ *j*, where S denotes the whole state space of the CTMC and $S^{\left(0\right)},S^{\left(1\right)},S^{\left(2\right)},\ldots$ denote the levels successively numbered by 0, 1, 2, …. Usually, one component of the state space is defined as the level number (see, e.g., [23–25, 27, 28, 31, 32] for more details on the level definition) and QBDs are commonly classified according to the dependence of transition rates on the level.

In the case of level independent QBDs, the transition rates do not depend on the process level and the corresponding generator matrices obey a structure of repeated identical blocks. The equilibrium distribution then has matrix-geometric form based on which efficient and numerically stable algorithms are available [26, 30–32]. However, for stochastic epidemics with demography level independent QBDs are not appropriate, because infection and recovery rates as well as birth rates and death rates of individuals should depend on the population size of the corresponding epidemiological class.

If the transition rates are allowed to depend on the level (transition rates are functions of the level), the process is referred to as a level-dependent QBD (LDQBD) process. Although they are more complicated to analyze than level independent QBDs, some notable matrix-analytic approaches for computing equilibrium distributions of LDQBDs exist [24, 27, 29]. In particular, the algorithm recently proposed by [24] provides an efficient and numerically stable means for this purpose. The art of LDQBD process construction is the appropriate choice of the level and obtaining the block matrices for specific models, which we shall investigate in the following two sections for our stochastic epidemics such that it becomes clear that LDQBDs are appropriate for stochastic epidemic modeling. Once an LDQBD process has been constructed, its equilibrium distribution can be efficiently obtained by matrix-analytic methods.

Hence, assume that we have constructed an LDQBD process such that the generator matrix of the CTMC is block tridiagonal, that is,

$$\begin{array}{c} Q=\left(\begin{array}{ccccc} {\tilde{Q}}_{00} & {\tilde{Q}}_{01} & & & \\ {\tilde{Q}}_{10} & {\tilde{Q}}_{11} & {\tilde{Q}}_{12} & & \\ & {\tilde{Q}}_{21} & {\tilde{Q}}_{22} & {\tilde{Q}}_{23} & \\ & & \ddots & \ddots & \ddots \end{array}\right) \end{array}$$ (1)

with blocks ${\tilde{Q}}_{mn}\in R^{d_{m}\times d_{n}}$, where *d*_*m*_ and *d*_*n*_ are the numbers of states in levels *m* and *n*, respectively, that is, $d_{m}=\left|S^{\left(m\right)}\right|$ and $d_{n}=\left|S^{\left(n\right)}\right|$. Note that the dimension of the state space must be finite, but it is allowed that the state space is infinite, which is the case as soon as one component is not upper bounded. We only require the above block tridiagonal matrix structure.

For an ergodic CTMC the equilibrium distribution *π* is the unique positive solution to *πQ* = 0 subject to the normalization condition π1=1. Now, let the equilibrium distribution *π* be partitioned compatibly with *Q*, that is, $\pi =({\tilde{\pi }}_{0},{\tilde{\pi }}_{1},{\tilde{\pi }}_{2}\;\ldots )$ with row subvectors ${\tilde{\pi }}_{n}\in R^{1\times d_{n}}$. Then *πQ* = 0 can be expressed as

$$\begin{array}{ccc} {\tilde{\pi }}_{0}{\tilde{Q}}_{00}+{\tilde{\pi }}_{1}{\tilde{Q}}_{10} & = & 0, \end{array}$$ (2)

$$\begin{array}{ccc} {\tilde{\pi }}_{n-1}{\tilde{Q}}_{n-1,n}+{\tilde{\pi }}_{n}{\tilde{Q}}_{nn}+{\tilde{\pi }}_{n+1}{\tilde{Q}}_{n+1,n} & = & 0,\;n>0. \end{array}$$ (3)

Inserting this into the recurrence scheme ${\tilde{\pi }}_{n+1}={\tilde{\pi }}_{n}R_{n},n\geq 0$, with nonnegative matrices $R_{n}\in R^{d_{n}\times d_{n+1}}$ whose entries are conditional sojourn times [27] we get

$$\begin{array}{ccc} {\tilde{\pi }}_{0}\left({\tilde{Q}}_{00}+R_{0}{\tilde{Q}}_{10}\right) & = & 0, \end{array}$$ (4)

$$\begin{array}{ccc} {\tilde{\pi }}_{n-1}\left({\tilde{Q}}_{n-1,n}+R_{n-1}Q_{nn}+R_{n-1}R_{n}{\tilde{Q}}_{n+1,n}\right) & = & 0,\;n>0, \end{array}$$ (5)

where

$$\begin{array}{c} R_{n-1}=-{\tilde{Q}}_{n-1,n}{\left({\tilde{Q}}_{nn}+R_{n}{\tilde{Q}}_{n+1,n}\right)}^{-1},\;n>0 \end{array}$$ (6)

can be computed recursively, given a suitable *R*_*N*_ for some finite *N* as a starting point. According to [24] one can set *R*_*N*_: = 0 for *N* large enough that the probability mass in levels higher than *N* is negligibly small. Then the generator matrix is truncated accordingly, without any augmentation necessary. This yields the following core algorithm for computing equilibrium distributions of LDQBDs:

- Choose *N* large and define $R_{N}=0\in R^{d_{N}\times d_{N+1}}$;
- For *n* = *N* − 1, *N* − 2, …, 0 compute $R_{n}=-{\tilde{Q}}_{n,n+1}{\left({\tilde{Q}}_{n+1,n+1}+R_{n+1}{\tilde{Q}}_{n+2,n+1}\right)}^{-1}$;
- Determine a nontrivial solution *x*_0_ ≠ 0 of $x_{0}\left({\tilde{Q}}_{00}+R_{0}{\tilde{Q}}_{10}\right)=0$;
- For *n* = 0, …, *N* − 1 compute *x*_*n*+1_ = *x*_*n*_ *R*_*n*_;
- By normalizing *x* = (*x*_0_, …, *x*_*N*_), determine $\pi =({\tilde{\pi }}_{0},{\tilde{\pi }}_{1},{\tilde{\pi }}_{2}\;\ldots )$, that is,
  $$\begin{array}{c} {\tilde{\pi }}_{n}=\frac{x_{n}}{\left|\right|x_{0}\left|\right|+\cdots +\left|\right|x_{N}\left|\right|}, \end{array}$$
  where ||⋅|| denotes the row sum norm.

Further details and a memory-efficient implementation are given in [24] where also the efficiency of the matrix-analytic computations is demonstrated.

### Generalized stochastic SI(S) models

In SI and SIS models the population consists of susceptibles and infectives. The state of the underlying CTMC is denoted by (*s*, *i*) where *s* is the number of susceptible individuals and *i* is the number of infected individuals. Both susceptibles and infectives can immigrate or can be born and they can die (or emigrate). Susceptibles can be infected and infectives can recover. With some constants we have the state transitions starting in (*s*, *i*) as given in Table 1.

**Table 1:** State transitions in generalized stochastic SI and SIS models.

Event	Next state	Transition rate
Birth/Immigration of susceptible	(*s* + 1, *i*)	λ_0_ *s* + *a*_0_
Birth/Immigration of infective	(*s*, *i* + 1)	λ_1_ *i* + *a*_1_
Death of susceptible	(*s* − 1, *i*)	*μ*_0_ *s*
Death of infective	(*s*, *i* − 1)	*μ*_1_ *i*
Infection of susceptible	(*s* − 1, *i* + 1)	$\beta_{0}\frac{s\cdot i}{s+i}$
Recovery of infective	(*s* + 1, *i* − 1)	*γ*_1_ *i*

Next state and corresponding transition rate when event occurs, given the current state is (*s*, *i*). For the SI model the recovery rate is zero, that is, *γ*_1_ = 0.

The infection rate $\beta_{0}\frac{s\cdot i}{s+i}$ is the product of a contact rate *β*_0_, the number of susceptible individuals *s*, and the proportion *i*/(*s* + *i*) of the population that is infected. If the recovery rate is chosen as *γ*_1_ = 0 we get a SI model, for *γ*_1_ > 0 we have a SIS model. Finally, the basic reproduction number of our model is given by $R_{0}=\frac{\beta_{0}}{\gamma_{1}+\mu_{1}}$. Note that the basic reproduction number is a fundamental concept originating from deterministic epidemic models where it is defined as the number of secondary infections caused by one infected individual in an entirely susceptible population, but the concept similarly applies to stochastic models with closed as well as open populations with fluctuating number of susceptibles, for details see, e.g., [9, 16, 17, 34, 35].

Now, we turn to modeling these stochastic epidemics by LDQBD processes. In order to get the desired block tridiagonal structure of the generator matrix we define the level number as the number of susceptibles and arrange the states in lexicographical order. In the present case, the number of infectives is not bounded by definition. Therefore, in order to get finite blocks we have to choose a truncation number denoted by *i*_max_, which can be safely set such that the probability of having more than *i*_max_ infectives is negligible. Doing so we have the state space $S=\left\{0,1,2,\ldots \right\}\times \left\{0,\ldots ,i_{\max}\right\}$. Since we have defined the level number as the number of susceptibles, which means for any state its first component defines the level number, it is clear that each level consists of *i*_max_ + 1 states, namely for level number *s*, corresponding to exactly *s* susceptibles, the level consists of all states where exactly *s* susceptibles are present, that is, $S^{\left(s\right)}=\left\{\left(s,0\right),\left(s,1\right),\ldots ,\left(s,i_{\max}+1\right)\right\}$. Hence, the generator matrix *Q* is of block tridiagonal structure with constant dimensions *d*_*i*_ = *i*_max_ + 1 and blocks

$$\begin{array}{ccc} {\tilde{Q}}_{n,n+1} & = & \left(\begin{array}{cccc} \lambda_{0}n+a_{0} & & & \\ \gamma_{1} & \lambda_{0}n+a_{0} & & \\ & 2\gamma_{1} & \lambda_{0}n+a_{0}\\ & \;\ddots & \;\ddots \\ & & i_{\max}\gamma_{1} & \lambda_{0}n+a_{0} \end{array}\right), \end{array}$$ (7)

$$\begin{array}{ccc} {\tilde{Q}}_{n,n-1} & = & \left(\begin{array}{ccccc} \mu_{0}n & \beta_{0}\frac{n\cdot 0}{n+0} & & & \\ & \mu_{0}n & \beta_{0}\frac{n\cdot 1}{n+1} & & \\ & & \mu_{0}n & \beta_{0}\frac{n\cdot 2}{n+2} & \\ & & \;\ddots & \;\ddots \\ & & & \mu_{0}n & \beta_{0}\frac{n\cdot (i_{\max}-1)}{n+i_{\max}-1}\\ & & & & \mu_{0}n \end{array}\right), \end{array}$$ (8)

$$\begin{array}{ccc} {\tilde{Q}}_{nn} & = & \left(\begin{array}{cccc} -\xi_{n,0} & a_{1} & \\ \mu_{1} & -\xi_{n,1} & \lambda_{1}+a_{1} & \\ \;\ddots & \;\ddots & \;\ddots & \\ & \left(i_{\max}-1\right)\mu_{1} & -\xi_{n,i_{\max}-1} & \left(i_{\max}-1\right)\lambda_{1}+a_{1}\\ & & i_{\max}\mu_{1} & -\xi_{n,i_{\max}} \end{array}\right), \end{array}$$ (9)

where the definitions for ${\tilde{Q}}_{n,n+1}$ and ${\tilde{Q}}_{nn}$ hold for *n* ≥ 0, the definition for ${\tilde{Q}}_{n,n-1}$ holds for *n* > 0, and *ξ*_*s*, *i*_ is defined as

$$\begin{array}{c} \xi_{s,i}=\lambda_{0}s+a_{0}+\lambda_{1}i+a_{1}+\mu_{0}s+\mu_{1}i+\beta_{0}\frac{si}{s+i}+\gamma_{1}i. \end{array}$$ (10)

For *s* = *i* = 0 the term $\frac{si}{s+i}$ is defined to be 0. Note that due to the truncation of the number of infectives not all row sums of *Q* need to be zero. If the entries of ${\tilde{Q}}_{ij}$ are denoted by ${\left({\tilde{Q}}_{ij}\right)}_{u,v}$ for *u* = 0, 1, …, *d*_*i*_ − 1 and *v* = 0, 1, …, *d*_*j*_ − 1, then we can also define the block matrices formally by all non-zero entries:

$$\begin{array}{ccc} {\left({\tilde{Q}}_{n,n+1}\right)}_{\left(u,v\right)} & = & \left\{\begin{array}{cc} \lambda_{0}n+a_{0}, & u=0,\ldots ,i_{\max},\;v=u,\\ \gamma_{1}u, & u=1,\ldots ,i_{\max},\;v=u-1, \end{array}\right. \end{array}$$ (11)

$$\begin{array}{ccc} {\left({\tilde{Q}}_{n,n-1}\right)}_{\left(u,v\right)} & = & \left\{\begin{array}{cc} \mu_{0}n, & u=0,\ldots ,i_{\max},\;v=u,\\ \beta_{0}\frac{nu}{n+u}, & u=0,\ldots ,i_{\max}-1,\;v=u+1, \end{array}\right. \end{array}$$ (12)

$$\begin{array}{ccc} {\left({\tilde{Q}}_{nn}\right)}_{\left(u,v\right)} & = & \left\{\begin{array}{cc} -\xi_{n,u}, & u=0,\ldots ,i_{\max},\;v=u,\\ \lambda_{1}u+a_{1}, & u=0,\ldots ,i_{\max}-1,\;v=u+1,\\ \mu_{1}u, & u=1,\ldots ,i_{\max},\;v=u-1. \end{array}\right. \end{array}$$ (13)

### Generalized stochastic SIR(S) models

In the SIR model infected individuals become immune (removals) after recovery. Hence, there is a third epidemiological class within the population and we denote by *r* the number of such immune removals. It is also possible that an immune individual, hence a removal, immigrates or is born into the population. In the SIRS model immunity can be lost and a removal loosing immunity becomes again a susceptible. As for the other epidemiological classes, immune individuals can be born, they can immigrate, die, or emigrate. Starting from state (*s*, *i*, *r*) we have the state transitions as given in Table 2 where for the SIR model the rate of loss of immunity is *γ*_2_ = 0 and for the SIRS model *γ*_2_ > 0. Note in particular, that now the state space of the underlying CTMC is three-dimensional. The basic reproduction number remains as for the SI(S) models.

**Table 2:** State transitions in generalized stochastic SIR and SIRS models.

Event	Next state	Transition rate
Birth/Immigration of susceptible	(*s* + 1, *i*, *r*)	λ_0_ *s* + *a*_0_
Birth/Immigration of infective	(*s*, *i* + 1, *r*)	λ_1_ *i* + *a*_1_
Birth/Immigration of removal	(*s*, *i*, *r* + 1)	λ_2_ *r* + *a*_2_
Death of susceptible	(*s* − 1, *i*, *r*)	*μ*_0_ *s*
Death of infective	(*s*, *i* − 1, *r*)	*μ*_1_ *i*
Death of removal	(*s*, *i*, *r* − 1)	*μ*_2_ *r*
Infection of susceptible	(*s* − 1, *i* + 1, *r*)	$\beta_{0}\cdot \frac{si}{s+i+r}$
Recovery from infection to immunity	(*s*, *i* − 1, *r* + 1)	*γ*_1_ *i*
Loss of immunity	(*s* + 1, *i*, *r* − 1)	*γ*_2_ *r*

Next state and corresponding transition rate when event occurs, given the current state is (*s*, *i*, *r*). For the SIR model *γ*_2_ = 0.

We proceed by showing how to model these stochastic epidemics appropriately as an LDQBD process. Again, we choose the level number to be the number *s* of susceptible individuals. In order to get finite blocks we truncate both the number of infectives and the number of removals, denoting the maximum numbers by *i*_max_ and *r*_max_, respectively. The block matrix dimensions *d*_*n*_ = (*i*_max_ + 1)(*r*_max_ + 1) are constant. We get

$$\begin{array}{c} {\tilde{Q}}_{n,n+1}=\left(\begin{array}{cccc} \Lambda \\ & \;\Lambda \\ & & \;\ddots \\ & & & \;\Lambda \end{array}\right)\in R^{\left(i_{\max}+1\right)\times \left(i_{\max}+1\right)} \end{array}$$ (14)

with *i*_max_ + 1 blocks

$$\begin{array}{c} \Lambda =\left(\begin{array}{cccc} \lambda_{0}n+a_{0}\\ \gamma_{2} & \lambda_{0}n+a_{0}\\ & 2\gamma_{2} & \lambda_{0}n+a_{0}\\ & \;\ddots & \;\ddots \\ & & r_{\max}\gamma_{2} & \lambda_{0}n+a_{0} \end{array}\right) \end{array}$$ (15)

and

$$\begin{array}{c} {\tilde{Q}}_{n,n-1}=\left(\begin{array}{ccccc} M & M_{0}\\ & M & M_{1}\\ & & \ddots & \ddots \\ & & & M & M_{i_{\max}-1}\\ & & & & M \end{array}\right) \end{array}$$ (16)

with blocks

$$\begin{array}{c} M=\text{diag}\left(\lambda_{0}n+a_{0}\right)\in R^{\left(r_{\max}+1\right)\times \left(r_{\max}+1\right)},\;M_{i}=\text{diag}{\left(\beta_{0}\frac{ni}{n+i+r}\right)}_{r=0}^{r_{\max}}. \end{array}$$ (17)

Finally,

$$\begin{array}{c} {\tilde{Q}}_{nn}=\left(\begin{array}{ccccc} N_{0} & N_{0}^{+}\\ N_{1}^{-} & N_{1} & N_{1}^{+}\\ & \ddots & \ddots & \ddots & \\ & & N_{i_{\max}-1}^{-} & N_{i_{\max}-1} & N_{i_{\max}-1}^{+}\\ & & & N_{i_{\max}}^{-} & N_{i_{\max}} \end{array}\right) \end{array}$$ (18)

with blocks

$$\begin{array}{ccc} N_{i} & = & \left(\begin{array}{cccc} -\xi_{n,i,0} & a_{2}\\ \mu_{2} & -\xi_{n,i,2} & \lambda_{2}+a_{2}\\ \;\ddots & \;\ddots & \;\ddots & \\ & \left(r_{\max}-1\right)\mu_{2} & \;-\xi_{n,i,r_{\max}-1}\; & \left(r_{\max}-1\right)\lambda_{2}+a_{2}\\ & & r_{\max}\mu_{2} & \;-\xi_{n,i,r_{\max}} \end{array}\right), \end{array}$$ (19)

$$\begin{array}{ccc} N_{i}^{-} & = & \left(\begin{array}{ccc} \mu_{1}i & \gamma_{1}i\\ \;\ddots & \;\ddots \\ & \mu_{1}i & \gamma_{1}i\\ & & \mu_{1}i \end{array}\right)\in R^{\left(r_{\max}+1\right)\times \left(r_{\max}+1\right)}, \end{array}$$ (20)

$$\begin{array}{ccc} N_{i}^{+} & = & \text{diag}\left(\lambda_{1}i+a_{1}\right)\in R^{\left(r_{\max}+1\right)\times \left(r_{\max}+1\right)}. \end{array}$$ (21)

For constructing the blocks $N_{i}$ in ${\tilde{Q}}_{nn}$ we use the definition

$$\begin{array}{c} \xi_{s,i,r}=\lambda_{0}s+a_{0}+\lambda_{1}i+a_{1}+\lambda_{2}r+a_{2}+\mu_{0}s+\mu_{0}i+\mu_{0}r+\beta_{0}\frac{si}{s+i+r}+\gamma_{1}i+\gamma_{2}r. \end{array}$$ (22)

## Results

In this section we present numerical examples in order to demonstrate that the LDQBD modeling formalism in conjunction with matrix-analytic solution methods is well-suited for studying the equilibrium distributions of the introduced stochastic epidemic models. Furthermore, once the equilibrium distributions are obtained, also moments and cumulants can be easily computed, in particular expectations and standard deviations are readily available. All results presented in the following tables and figures are numerical solutions of the LDQBD models obtained via the matrix-analytic solution method, avoiding the use of costly stochastic simulations. The suitability of matrix analytic solution methods for LDQBD models has been explained in the Methods section and is well known from numerous previous applications [23–25, 27, 28]. In particular, for further details on the suitability and accuracy of the state space truncation we refer to [24, 27, 36]. What indeed allows us to apply matrix-analytic methods is that we have managed to model the considered stochastic epidemics with immigration and demographic effects by LDQBD processes, which is therefore a key contribution.

Clearly, we have to restrict the following presentation to selected parameters. We first study for SI(S) the impact of varying immigration rates on the equilibrium distributions of the epidemiological classes. Of course, we can also study varying contact rates, recovery rates, birth rates, and death rates, but as immigration of susceptibles and infectives is one distinguishing feature of our models we shall focus on the respective rates. Furthermore, we study for SIR(S) the impact of the immunity loss rate on the respective equilibrium distributions. In all cases, appropriate truncation points as explained before have been chosen where checking the sum of all absolute values of *πQ* with the truncated generator matrix assures that indeed no significant probability mass is assigned to the truncated portion of the state space. For the remaining states with significant probability all joint equilibrium probabilities are computed, that is, the whole equilibrium distribution. These computations are very efficient, only a few seconds per parameter setting are required.

### Varying immigration rates in generalized stochastic SI(S) models

For numerical examples of the SI and SIS models we consider as a starting point the basic parameter setting *β*_0_ = 30, λ_0_ = 5, λ_1_ = 5, *γ*_1_ = 5, *μ*_0_ = 4, *μ*_1_ = 15, which yields the basic reproduction number $R_{0}=\frac{30}{20}=1.5$. The parameters are chosen so that for a reasonable basic reproduction number the system/model dynamics in terms of transition rates (some of which in addition to the above parameters also depend on the current numbers of individuals of certain epidemiological classes) related to the demography and the epidemic, respectively, are of the same order of magnitude and on the same time scale. Starting from this parameter setting, equilibrium distributions are computed for different values of the immigration rates *a*_0_ and *a*_1_ of susceptibles and infectives.

From the joint equilibrium probabilities one easily gets marginal equilibrium probabilities of the numbers of susceptibles and infectives, respectively, as well as equilibrium probabilities of the total population size, and the proportion of infectives within the total population, that is the ratio of the number of infectives to the total population size.

**Fig 1:**
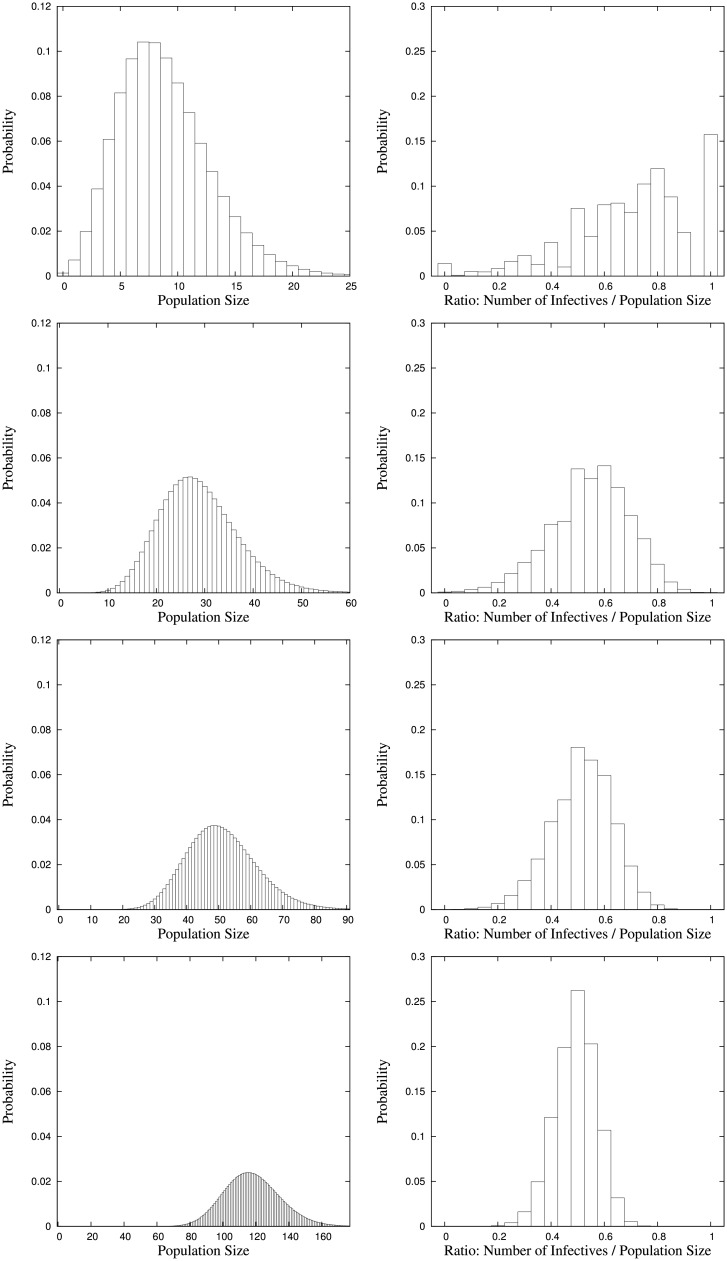
Equilibrium distributions of the total population size and the ratio of the number of infectives to the total population size for parameter setting *a*_1_ = 50, *β*_0_ = 30, λ_0_ = 5, λ_1_ = 5, *γ*_1_ = 5, *μ*_0_ = 4, *μ*_1_ = 15 with immigration rates of susceptibles *a*_0_ = 10, 100, 200, 500 (top to bottom).

**Fig 2:**
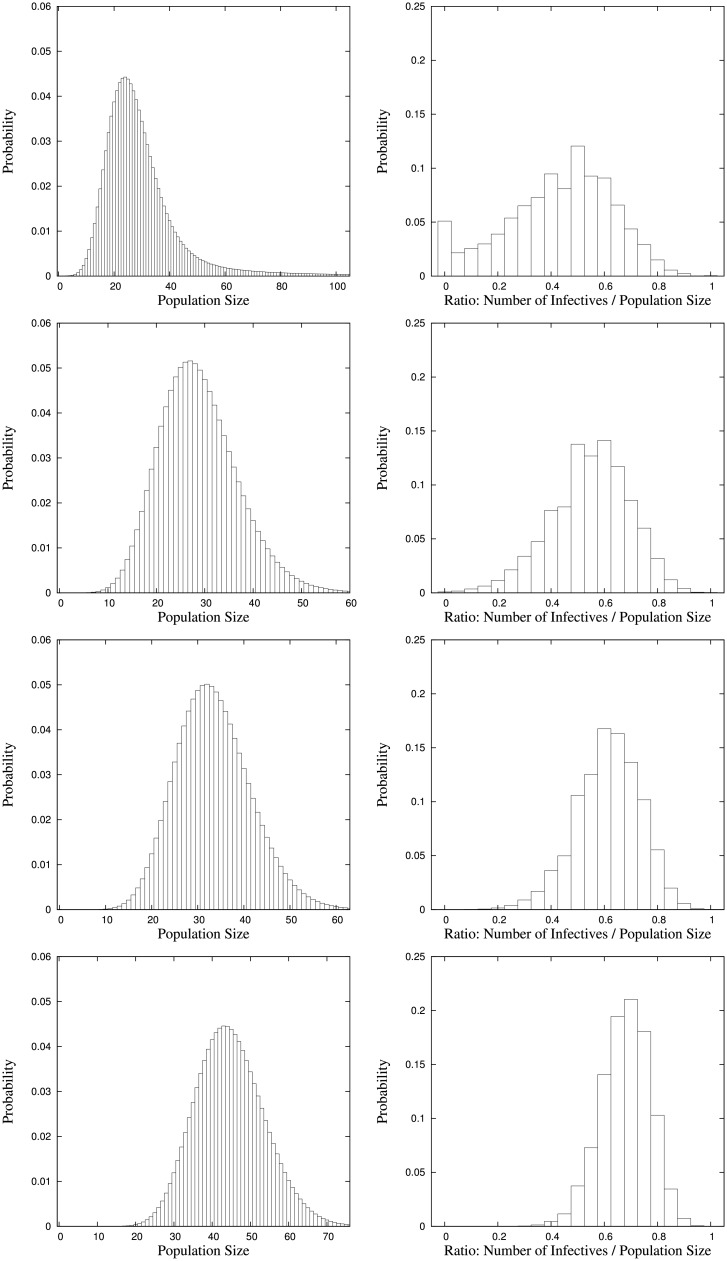
Equilibrium distributions of the total population size and the ratio of the number of infectives to the total population size for parameter setting *a*_0_ = 100, *β*_0_ = 30, λ_0_ = 5, λ_1_ = 5, *γ*_1_ = 5, *μ*_0_ = 4, *μ*_1_ = 15 with immigration rates of infectives *a*_1_ = 10, 50, 100, 200 (top to bottom).

In order to keep the presentation of equilibrium distributions well-arranged and not become too excessive we focus on the equilibrium distributions of the total population size and on the proportion of infectives. These are depicted for the different parameter settings in Figs 1 and 2 where each figure contains the equilibrium distributions for four different values of the respective immigration rate that is varied. Expectations and standard deviations of the equilibrium numbers of susceptibles, infectives and the population size are given in Tables 3 and 4.

**Table 3:** Expectations and standard deviations of the equilibrium numbers of susceptibles, infectives and the population size for parameter setting *a*_1_ = 50, *β*_0_ = 30, λ_0_ = 5, λ_1_ = 5, *γ*_1_ = 5, *μ*_0_ = 4, *μ*_1_ = 15 with immigration rates of susceptibles *a*_0_ = 10, 100, 200, 500.

	*a*_0_ = 10		*a*_0_ = 100		*a*_0_ = 200		*a*_0_ = 500	
	Expect	StdDev	Expect	StdDev	Expect	StdDev	Expect	StdDev
Susceptibles	2.577	2.299	12.547	5.988	23.675	8.387	57.004	13.067
Infectives	6.258	3.300	16.255	5.917	27.367	7.881	60.700	11.952
Population Size	8.834	4.037	28.801	8.147	51.043	11.060	117.704	16.944

**Table 4:** Expectations and standard deviations of the equilibrium numbers of susceptibles, infectives and the population size for parameter setting parameter setting *a*_0_ = 100, *β*_0_ = 30, λ_0_ = 5, λ_1_ = 5, *γ*_1_ = 5, *μ*_0_ = 4, *μ*_1_ = 15 with immigration rates of infectives *a*_1_ = 10, 50, 100, 200.

	*a*_1_ = 10		*a*_1_ = 50		*a*_1_ = 100		*a*_1_ = 200	
	Expect	StdDev	Expect	StdDev	Expect	StdDev	Expect	StdDev
Susceptibles	18.933	23.981	12.547	5.988	12.198	5.165	13.356	5.010
Infectives	12.726	7.798	16.255	5.917	21.220	6.294	31.336	7.315
Population Size	31.659	24.968	28.801	8.147	33.417	8.110	44.692	9.033

Fig 1 shows the equilibrium distributions for the different values *a*_0_ = 10, *a*_0_ = 100, *a*_0_ = 200, and *a*_0_ = 500 of the immigration rate of susceptibles, while the other parameters are fixed. As can be seen, with increasing immigration rate of susceptibles the proportion of infectives decreases and the equilibrium distribution becomes smoother and smoother (more regular). Furthermore, both the number of susceptibles and infectives increase, thus, also the total population size. Interpretations are as follows. Clearly, the immigration rate of susceptibles directly impacts on the number of susceptibles. Since these of course can be infected, the number of infectives increases, too. But the increase in the number of infectives is less strong than that of susceptibles such that the proportion of infectives within the total population decreases. The equilibrium distribution of the proportion of infectives is rather irregular for small *a*_0_, as then the total population size is rather small and for instance the ratio/proportion has a peak at one half. For increasing immigration rate of susceptibles and accordingly larger total population sizes this effect becomes weaker and weaker.

The equilibrium distributions for the values *a*_1_ = 10, *a*_1_ = 50, *a*_1_ = 100, and *a*_1_ = 200 of the immigration rate of infectives with the other parameters kept fixed are depicted in Fig 2. Here, we observe an increasing proportion of infectives and a slightly increasing total population. This can be explained due to the facts that infectives die at a higher rate than susceptibles and that the infection rate is *β*_0_*si*/(*s* + *i*). Therefore, with a small immigration rate of infectives, the probability of very few infectives is quite large, only very few become infected. In contrast, with higher immigration rate of infectives, also more members of the population can become infected such that the proportion of infectives increases, while the total population size increases only slightly because of the higher death rate of infectives.

### Varying immunity loss rates in generalized stochastic SIR(S) models

Now, we turn to the study of SIR(S) models where we focus on varying immunity loss rates, which is a distinguishing feature as compared to SI(S) models. Actually, we have also studied varying immigration rates and observed similar effects as for SI(S) models, which are therefore omitted here. We consider the different values *γ*_2_ = 0, *γ*_2_ = 5, *γ*_2_ = 10 and *γ*_2_ = 30 of the immunity loss rates, while the other parameters are fixed as *a*_0_ = 100, *a*_1_ = 50, *a*_2_ = 0, *β*_0_ = 30, λ_0_ = λ_1_ = λ_2_ = 5, *μ*_0_ = *μ*_2_ = 6, *μ*_1_ = 15, *γ*_1_ = 5. Hence, the basic reproduction number is $R_{0}=\frac{30}{20}=1.5$, as before in the SI(S) models. Note that in general our model framework allows immigration of removals, too, but here according to *a*_2_ = 0 we do not consider immigration of removals.

**Fig 3:**
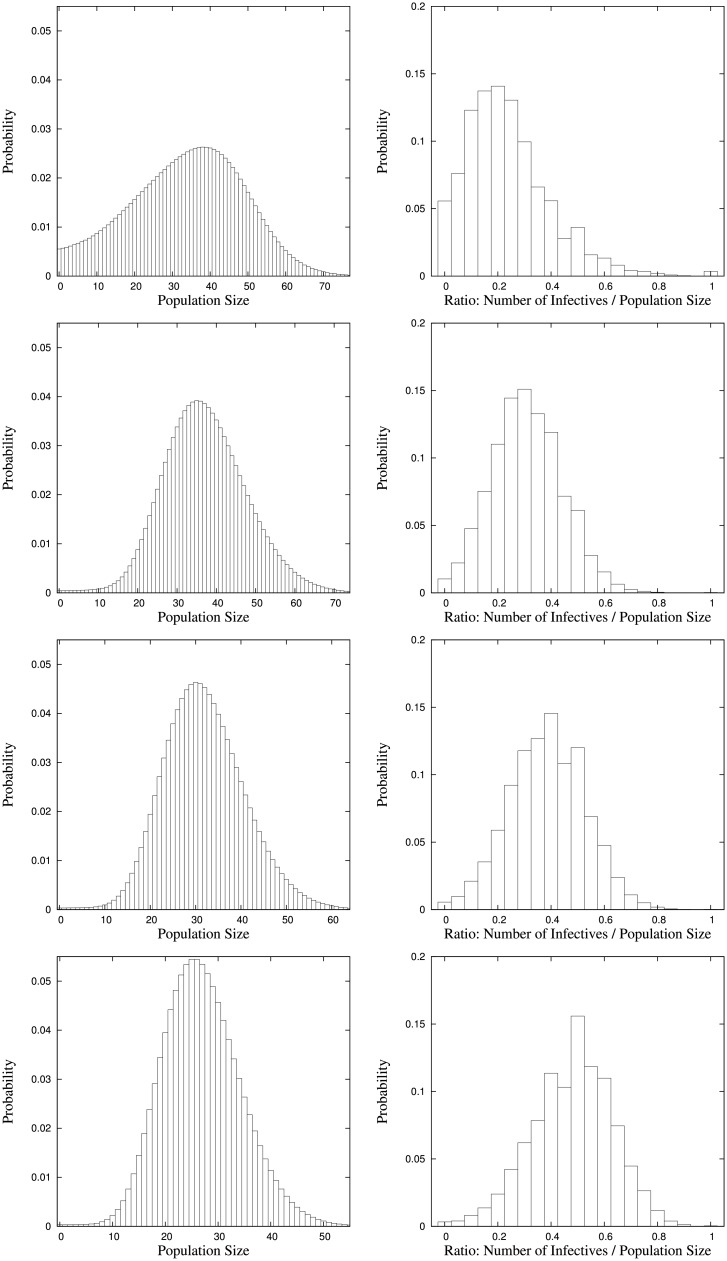
Equilibrium distributions of the total population size and the ratio of the number of infectives to the total population size for parameter setting *a*_0_ = 100, *a*_1_ = 50, *a*_2_ = 0, *β*_0_ = 30, λ_0_ = λ_1_ = λ_2_ = 5, *μ*_0_ = *μ*_2_ = 6, *μ*_1_ = 15, *γ*_1_ = 5 with immunity loss rates *γ*_2_ = 0, 5, 10, 30 (top to bottom).

**Fig 4:**
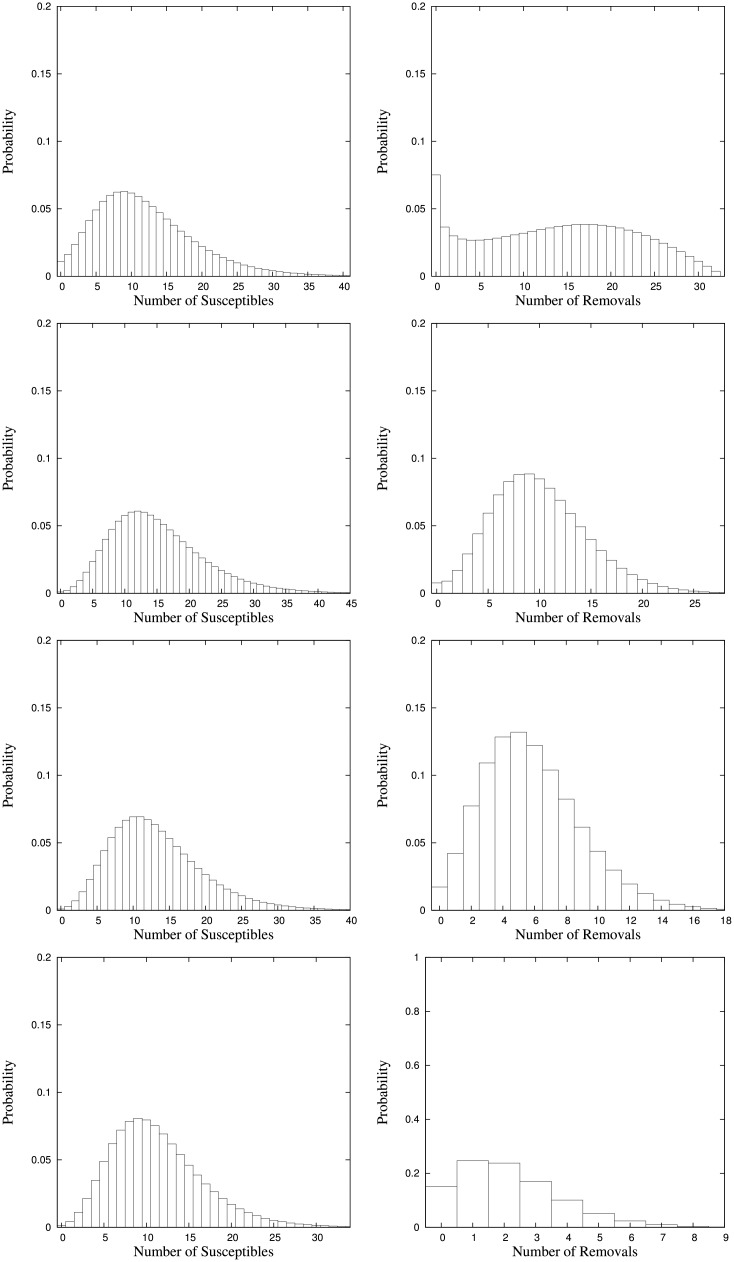
Equilibrium distributions of the number of susceptibles and the number of removals for parameter setting *a*_0_ = 100, *a*_1_ = 50, *a*_2_ = 0, *β*_0_ = 30, λ_0_ = λ_1_ = λ_2_ = 5, *μ*_0_ = *μ*_2_ = 6, *μ*_1_ = 15, *γ*_1_ = 5 with immunity loss rates *γ*_2_ = 0, 5, 10, 30 (top to bottom).

The expectations and standard deviations of the equilibrium numbers of all epidemiological classes and the population size are given in Table 5. Fig 3 shows the equilibrium distributions of the total population size and the proportion of infectives and Fig 4 those of the numbers of susceptibles and removals. Here, in contrast to the SI(S) examples, we have added the latter because of a striking effect that appears in the case of *γ*_2_ = 0 (corresponding to SIR rather than to SIRS).

**Table 5:** Expectations and standard deviations of the equilibrium numbers of all epidemiological classes and the population size for parameter setting *a*_0_ = 100, *a*_1_ = 50, *a*_2_ = 0, *β*_0_ = 30, λ_0_ = λ_1_ = λ_2_ = 5, *μ*_0_ = *μ*_2_ = 6, *μ*_1_ = 15, *γ*_1_ = 5 with immunity loss rates *γ*_2_ = 0, 5, 10, 30.

	*γ*_2_ = 0		*γ*_2_ = 5		*γ*_2_ = 10		*γ*_2_ = 30	
	Expect	StdDev	Expect	StdDev	Expect	StdDev	Expect	StdDev
Susceptibles	12.029	7.120	15.138	7.299	13.269	6.453	11.358	5.514
Infectives	7.888	4.560	12.110	5.335	12.837	5.317	13.431	5.265
Removals	13.858	8.715	9.957	4.659	5.808	3.139	2.163	1.662
Population Size	33.775	14.810	37.204	10.563	31.915	8.919	26.952	7.539

Let us start with the cases in which the immunity loss rate is *γ*_2_ > 0. Here, we observe that with increasing immunity loss rate the numbers of susceptibles and removals as well as the total population size decrease whereas the number of infectives even slightly increases and thus the proportion of infectives increases. This effect is intuitively appealing and can be readily explained. If the immunity loss rate grows removals become again susceptibles at a growing rate. Therefore, the number of removals decreases. On the other hand, due to the form of the infection rate the number of susceptibles does not increase but decrease because the effective rate of infection grows such that the increasing immunity loss rate does not result in a larger number of susceptibles but in a larger number of infectives. Finally, since infectives die at a higher rate than susceptibles and removals, the overall population size decreases and due to an increase in the absolute number of infectives the proportion of infectives increases even more.

Now, for the SIR model (corresponding to *γ*_2_ = 0) recognize that compared to the cases where *γ*_2_ > 0, in particular the equilibrium distributions of the total population size and the number of removals show that there is a relatively large probability mass for very few and even zero population, which seems counterintuitive at a first glance. So, how can this be explained?

Consider the dynamics of the removals. If immunity cannot be lost then, since there is no immigration of removals, the population of removals can only change by births, deaths, and recovery from infection. If only births and deaths would be possible, then the population of removals would be fully described by a linear birth-and-death process with state-dependent birth rates λ_2_ *r* and state-dependent death rates *μ*_2_ *r* where *r* is the number of removals corresponding to the state of the linear birth-and-death process. It is well known that for such a linear birth-and-death process with λ_2_ < *μ*_2_ the equilibrium distribution is concentrated on zero. Thus, if recovery of infection is excluded then with probability one the equilibrium number of removals is zero. However, as in SIR(S) the population of removals is also fed by recovered infectives, the removals do not die out. Rather the recovery of infectives can be taken as a kind of immigration of infectives to the population of removals where the immigration rate is linear in the number of infectives which itself depends on the number of susceptibles and the infection rate.

In conjunction this explains the effect observed. The population of removals is described by a mixture of a linear birth-and-death process with an additional immigration processes that is independent of the number of removals. In effect, there is a peak for the probability of having no removals. But recognize that this peak is smaller than the peaks for larger numbers of removals in the cases where *γ*_2_ > 0. The mixture with being fed by recovered infectives results in a very flat equilibrium distribution of the number of removals having a relatively wide range of population values with significant equilibrium probability and quite large standard deviation, or variance, respectively.

In a similar manner the shape of the equilibrium distribution of the number of susceptibles can be explained. With *γ*_2_ = 0, there is no ‘cycle’ in the sense that individuals, once infected, can never become again susceptibles. Either they die as infective or become removals and eventually die as removal. Therefore, the number of susceptibles and thus the overall population size tends to be lower than for *γ*_2_ = 5.

## Discussion

Stochastic epidemics with immigration and demographic effects have been introduced where the corresponding stochastic processes (multi-dimensional continuous-time Markov chains) are non-absorbing and possess equilibrium probability distributions. Births, deaths, immigration, and emigration of individuals from all involved epidemiological classes are possible. In particular, the epidemic never dies out forever, that is, even when temporarily no infected individuals are present in the population, due to the possibility of immigration of new infectives from outside the epidemic can emerge again. This is reflected by constant population-size-independent immigration rates as part of combined immigration/birth rates.

We have shown how the multi-dimensional state spaces can be arranged such that with an appropriate numbering of states the models are level-dependent quasi-birth-and-death processes, that is, continuous-time Markov chains with block tridiagonal generator matrices. The model representations as LDQBD processes are particularly useful for efficient computation of equilibrium distributions using matrix-analytic methods.

Numerical examples for various parameter settings have been provided in order to demonstrate that this approach is valuable for gaining insights into a wide range of models with specific parameter settings. Since we have given general model formulations this can be taken as a general framework for studying diverse epidemics by choosing concrete parameters for the concrete epidemic under investigation.

Considering the type of non-absorbing models presented in this paper is very important, because these models realistically reflect that in many practical cases open sub-communities with immigration of individuals of all relevant epidemiological classes rather than closed global communities need to be studied. The LDQBD modeling approach in combination with matrix-analytic solution methods provides a general framework for such studies.

Further research is concerned with including even more features into the type of non-absorbing models, thereby broadening the class of models that yield to analysis by matrix-analytic methods. For instance, incorporating latent periods seems to be straightforward and yields models of SEIR(S) type. It is also possible to generalize the type of state dependence of the rates to any reasonable function of the system state. This does not destroy the block tridiagonality of the generator matrix and thus the LDQBD process structure is preserved. Also aging effects might be incorporated.

Another topic of further research is to consider alternative level definitions. In the present paper we defined one component of the state (the number of susceptibles) as the level number. Essentially due to this choice, in addition to the usual truncation of the state space at some high level number we need to truncate also within the blocks at sufficiently high numbers of infectives and removals, respectively. This does not seriously derogate the computational results, because no significant probability mass is truncated, but it might be even completely avoided. Because there is no formal requirement to choose one single component as the level, one might define the level via combining information from multiple components. More formally, the level might be defined by a function of the whole state rather than one component, for instance as the maximum or the sum of the state components. Very recently, similar ideas have been successfully applied in the context of stochastic chemical kinetics [28] and it is likely that in this manner the studies of stochastic epidemics by LDQBD processes can be further improved. Clearly, it is interesting to study how such alternative level definitions affect the resulting LDQBD structure as well as the computational effort.
